# The Naples Prognostic Score as a Predictor of High-Risk Coronary Plaques Detected on Coronary CT Angiography in Chronic Coronary Syndrome

**DOI:** 10.3390/jcm14082661

**Published:** 2025-04-13

**Authors:** Cagatay Bolgen, Mustafa Mazıcan

**Affiliations:** 1 Interventional Radiology Department, Adana Medline Hospital, 01170 Adana, Turkey; 2 Interventional Radiology Department, Adana Dr. Turgut Noyan Application and Research Center, Başkent University, 01250 Adana, Turkey; m_mazican@yahoo.com

**Keywords:** Naples Prognostic Score, high-risk coronary plaque, chronic coronary syndrome, revascularization, coronary CT angiography

## Abstract

**Background/Objectives:** High-risk coronary plaques (HRP), identified through coronary CT angiography (CCTA), are closely linked to cardiovascular events. Nutritional status and systemic inflammation may play a critical role in the development of HRP. The Naples Prognostic Score (NPS), which integrates markers of nutritional status and systemic inflammation, has emerged as a potential predictor of outcomes in various cardiovascular conditions. This study aimed to investigate the association between NPS and HRP as assessed by CCTA. **Methods:** A retrospective analysis was performed on 753 patients with chronic coronary syndrome (CCS) who underwent CCTA. The patients were categorized into two groups: those with high-risk plaques (HRP present), and those without (HRP absent). Additionally, they were further stratified based on their NPS. Univariable and multivariable logistic regression analyses were conducted to identify the most relevant clinical factors and the role of NPS in relation to HRP and the need for revascularization. **Results:** The study population had a mean age of 56.9 ± 10.7 years, with 40% being female. The NPS was significantly higher in the HRP-present group compared to the HRP-absent group (*p* = 0.001). Stratification by NPS groups revealed that higher NPS groups were associated with increased coronary artery calcification scores (CAC) and revascularization rates (*p* < 0.001 and *p* = 0.003, respectively). Multivariable regression analysis demonstrated a significant association between NPS and HRP (OR = 1.228, 95% CI: 1.013–1.489, *p* = 0.036). **Conclusions:** The NPS is independently associated with the presence of high-risk coronary plaques in patients with chronic coronary syndrome. NPS may serve as a complementary risk stratification tool by reflecting systemic inflammation and nutritional status. Further prospective studies are needed to validate its prognostic value.

## 1. Introduction

High-risk coronary plaques (HRP) play a pivotal role in predicting adverse cardiovascular events, which are detected by coronary computed tomography angiography (CCTA). These plaques, characterized by their vulnerability to rupture, represent a critical focus in the assessment and management of patients with chronic coronary syndrome (CCS) [1,2,3,4]. While well-established cardiovascular risk factors contribute to plaque formation, emerging evidence suggests that systemic inflammation and nutritional status also significantly influence the development of HRP [5].

The Naples Prognostic Score (NPS) is a composite index integrating parameters of nutritional status and systemic inflammation. It was originally developed as a prognostic tool in oncological settings. Recent studies have expanded its utility to various cardiovascular conditions, linking the score to patient outcomes in acute and chronic settings [6,7,8]. However, the association between NPS and HRP, particularly in the context of CCTA, remains underexplored. Understanding this relationship could bring valuable observations into the interaction between inflammation, nutrition, and coronary plaque vulnerability, potentially enhancing risk stratification and guiding clinical decision-making.

This study aims to investigate the association between NPS and HRP in patients with CCS undergoing CCTA. By evaluating the predictive value of NPS for HRP and its role in determining the need for revascularization, this research seeks to uncover novel pathways to optimize cardiovascular risk assessment and management strategies.

## 2. Materials and Methods

### 2.1. Study Population

**Figure 1:**
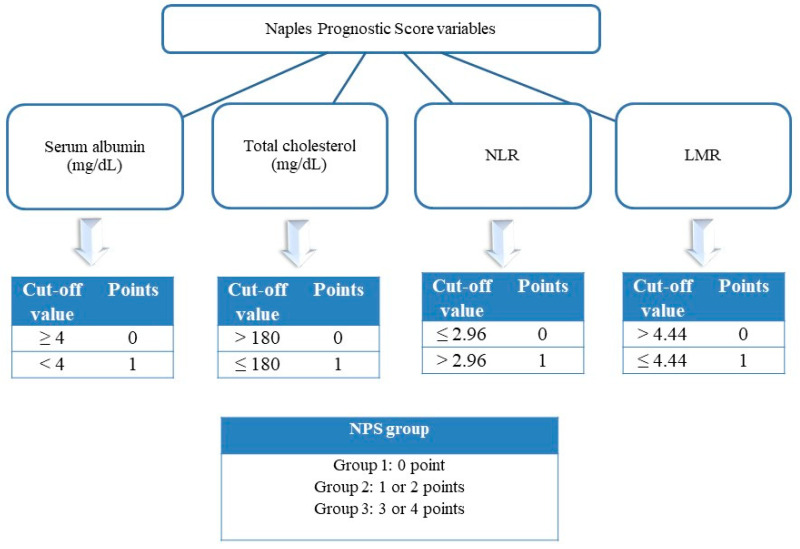
Classification of the Naples Prognostic Score (NPS), which combines serum albumin, total cholesterol, neutrophil-to-lymphocyte ratio (NLR), and lymphocyte-to-monocyte ratio (LMR). Patients are grouped into three risk categories: NPS 0 (Group 1), NPS 1–2 (Group 2), and NPS 3–4 (Group 3). This composite score reflects the interplay between nutritional status and systemic inflammation, and its association with coronary plaque vulnerability is investigated in this study.

The study population comprised 804 patients with suspected chronic coronary syndrome and low or moderate (>5–50%) pre-test likelihood of obstructive coronary artery disease (CAD) [9] who had undergone a CCTA between January 2022 and November 2024. Patients were excluded if they had suboptimal imaging quality (*n* = 2) or coexisting conditions such as previously diagnosed coronary artery disease, malignancies, inflammatory disorders, thyroid abnormalities, chronic liver disease, or end-stage renal disease (*n* = 15). Additionally, individuals receiving lipid-lowering therapy at baseline were excluded to prevent potential interference with the calculation of the NPS (*n* = 34). Following the exclusion process, a total of 753 patients were included. Firstly, the patients were categorized into two groups according to HRP presence. Subsequently, they were classified into three groups in accordance with the NPS. Data on demographic and clinical characteristics, laboratory findings, medication use, and revascularization status were retrieved from the hospital’s database. The NPS was calculated using four parameters: serum total cholesterol, albumin, lymphocyte-to-monocyte ratio, and neutrophil-to-lymphocyte ratio [10]. Based on their scores, patients were classified into three categories: Group 1 (NPS = 0), Group 2 (NPS = 1–2), and Group 3 (NPS = 3–4) (Figure 1). This study was carried out in compliance with the ethical principles outlined in the Declaration of Helsinki. The research protocol received approval from the local institutional ethics committee (Approval No: 01, dated 10 January 2025), and written informed consent was obtained from all participants in accordance with institutional review board guidelines.

### 2.2. Coronary Computed Tomography Angiography and Invasive Coronary Angiography

Coronary CT angiography was conducted using a 64-slice Optima CT660 scanner (GE Healthcare, Milwaukee, WI, USA) during which 75 mL of iodinated contrast agent (iobitridol, Xenetix 350, 350 mg/mL; Guerbet, Villepinte, France) was administered, followed by a 35 mL saline flush. Both were delivered through an 18-gauge cannula inserted into an antecubital vein at a rate of 5.5 mL/s. The electrocardiogram-gated coronary calcium scoring was performed using prospective gating with a collimation of 4 × 3.0 mm and a slice thickness of 3 mm. Scanning parameters included a tube current of 300 mA, tube voltage of 120 kV, and a gantry rotation time of 0.4 s. CACS were computed using Smart Score 4.0 software (GE Healthcare). Scanning was performed in a craniocaudal direction during a single mid-inspiratory breath-hold, with ECG gating applied. Image data were processed on specialized workstations (GE Volume Viewer 4.6; GE Healthcare), and reconstructions were generated at the optimal cardiac phase using a slice thickness of 0.625 mm and an increment of 0.3 mm. The quality of the coronary CT angiograms was assessed to ensure diagnostic adequacy.

**Figure 2:**
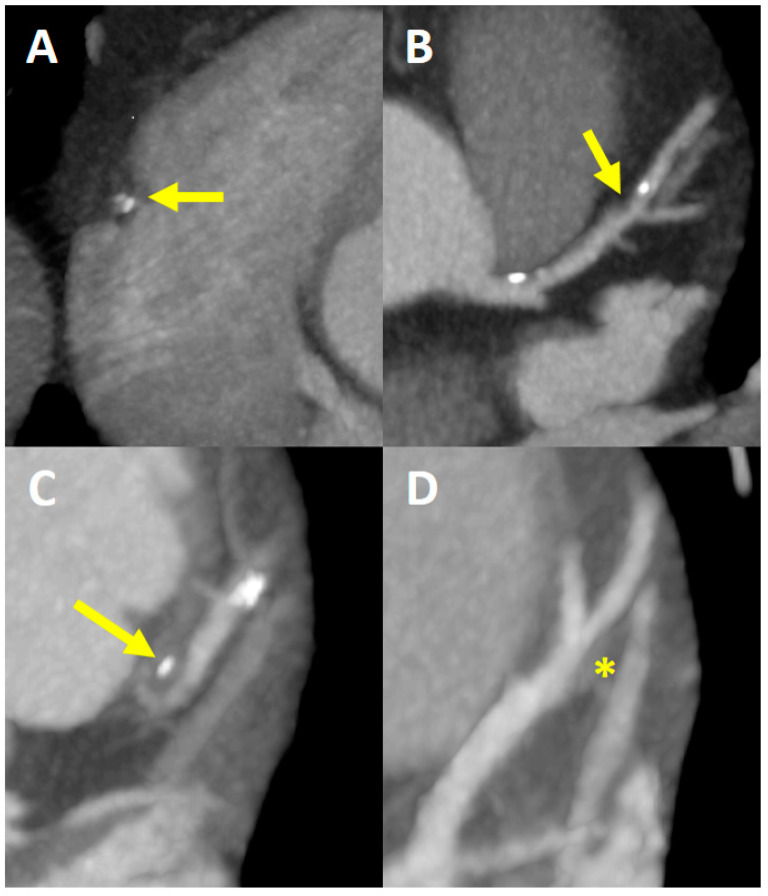
Representative images of high-risk plaque features on coronary computed tomography angiography (CCTA), including the napkin-ring sign ((**A**) with arrow), low-attenuation plaque (≤30 HU) ((**B**) with arrow), spotty calcification (<3 mm) ((**C**) with arrow), and positive remodeling (remodeling index ≥ 1.1) ((**D**) with asterisk). These imaging characteristics are used to define high-risk coronary plaques (HRP) in this study and serve as the primary outcome linked to NPS.

Coronary artery calcium was quantified using the Agatston method, which identifies calcified lesions as areas exceeding 1 mm^2^ in size with peak attenuation values above 130 Hounsfield Units (HU). These lesions were automatically detected and color-coded by the imaging software, and the total CACS was computed as the sum of all such lesions. HRP characteristics were defined as follows: low-attenuation plaque volume (LAP) with attenuation ≤ 30 HU, positive remodeling (remodeling index ≥ 1.1), spotty calcification (focal deposits < 3 mm in size), and the presence of a napkin-ring sign—characterized by a central low-attenuation area surrounded by a higher-attenuation rim (Figure 2) [11]. All scans were performed while patients were in sinus rhythm, and beta-blockers were administered when resting heart rates exceeded 60 beats per minute to enhance image quality.

Invasive coronary angiography was conducted in cases where non-invasive evaluation indicated a high risk of adverse events—for example, when CCTA revealed ≥50% stenosis in the left main coronary artery, ≥70% stenosis in the proximal left anterior descending artery with single- or two-vessel disease, or ≥70% stenosis in the proximal segments of all three major coronary arteries. Decisions regarding revascularization were made in accordance with current clinical guidelines [9].

### 2.3. Statistical Analysis

All statistical analyses were performed using SPSS software version 21.0 (SPSS Inc., Chicago, IL, USA). The distribution of continuous variables was assessed using the Kolmogorov–Smirnov test. Normally distributed variables were expressed as mean ± standard deviation (SD), while non-normally distributed variables were presented as median with interquartile range (IQR). Categorical variables were summarized as frequencies and percentages. Comparisons between two groups were conducted using the Independent Samples *t*-test for normally distributed variables and the Mann–Whitney U test for non-normally distributed data. For comparisons involving more than two groups, one-way ANOVA was used for normally distributed variables, and the Kruskal–Wallis test was applied to non-normally distributed variables. The Chi-square or Fisher’s exact test was used to analyze categorical variables, as appropriate. Correlation between continuous variables was assessed using Pearson’s correlation coefficient for normally distributed data and Spearman’s rank correlation coefficient for non-normally distributed data. Multicollinearity was evaluated using correlation matrices and variance inflation factors (VIF), with a VIF threshold of <2.0 indicating acceptable collinearity. Univariable logistic regression analysis was performed to identify factors associated with HRP and revascularization. Variables with a *p*-value < 0.10 in univariable analysis and those deemed clinically relevant were included in the multivariable logistic regression models to determine independent predictors. The discriminatory performance of the final multivariable models was assessed using receiver operating characteristic (ROC) curve analysis, with area under the curve (AUC) values and 95% confidence intervals (CI) reported. A two-sided *p*-value < 0.05 was considered statistically significant.

## 3. Results

**Figure 3:**
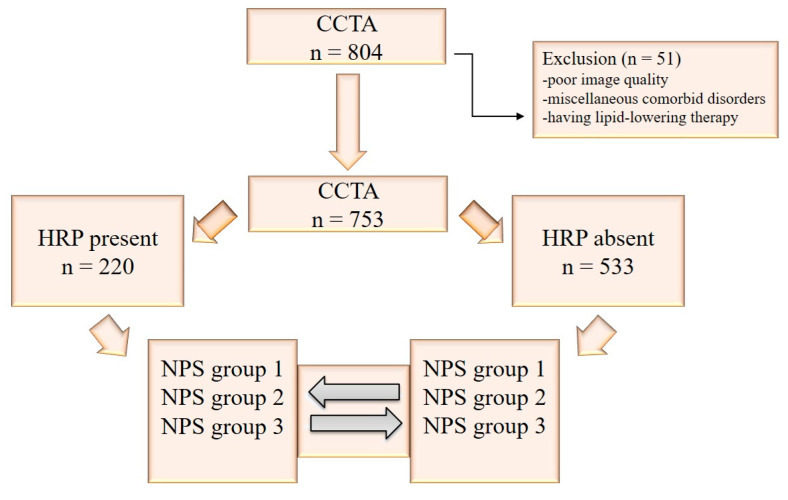
Study flow diagram (CCTA = coronary CT angiography, HRP = high-risk coronary plaques, NPS = Naples Prognostic Score).

Of 804 patients evaluated for eligibility, 51 were excluded. The study included 753 patients with a mean age of 56.9 ± 10.7 years, of whom 40% were female. Figure 3 shows the flow diagram.

Patients were stratified based on the presence of HRP, with significant differences observed between groups. The HRP-present group was older (61.7 ± 9.9 vs. 54.9 ± 10.3 years, *p* < 0.001) and had a lower prevalence of female patients (*p* < 0.001). Hypertension and dyslipidemia were more common in the HRP-present group (*p* = 0.001 and *p* = 0.015, respectively), while fasting blood glucose (102 [74–372] vs. 98 [67–396] mg/dL, *p* = 0.002), creatinine (0.9 [0.5–1.8] vs. 0.8 [0.5–1.3] mg/dL, *p* < 0.001), and HDL-C levels (42 [17–86] vs. 44 [22–95] mg/dL, *p* < 0.001) differed significantly between groups. Albumin levels were lower in the HRP-present group (4.1 ± 0.3 vs. 4.2 ± 0.2 g/dL, *p* < 0.001), and revascularization was significantly more frequent (49.5% vs. 1.9%, *p* < 0.001). The NPS was significantly higher in the HRP-present group (1.5 ± 0.9 vs. 1.2 ± 0.9, *p* = 0.001) with a notable difference in its distribution (*p* = 0.004) (Table 1).

Stratification by NPS showed increasing age (*p* = 0.001), hypertension prevalence (*p* = 0.014), and worsening lipid profile across higher NPS groups (*p* < 0.001 for total cholesterol, LDL-C, HDL-C, and triglycerides). Hemoglobin (*p* = 0.004), platelet count (*p* = 0.042), and albumin levels (*p* < 0.001) were lowest in the highest NPS group, whereas white blood cell count and CRP were highest (both *p* = 0.021). The coronary artery calcium (CAC) score was significantly greater in patients with higher NPS (*p* < 0.001), and revascularization rates increased across NPS groups (12.5%, 14.7%, and 29.3%, respectively; *p* = 0.003) (Table 2).

Multivariable regression analysis identified age (OR = 1.056, 95% CI: 1.038–1.075, *p* < 0.001), dyslipidemia (OR = 1.831, 95% CI: 1.052–3.185, *p* = 0.032), diabetes mellitus (OR = 1.788, 95% CI: 1.051–3.040, *p* = 0.032), glucose (OR = 1.008, 95% CI: 1.003–1.013, *p* = 0.001), creatinine (OR = 11.005, 95% CI: 3.734–32.432, *p* < 0.001), and NPS (OR = 1.228, 95% CI: 1.013–1.489, *p* = 0.036) as independent predictors of HRP, while age (OR = 1.050, 95% CI: 1.026–1.074, *p* < 0.001), dyslipidemia (OR = 2.787, 95% CI: 1.505–5.161, *p* = 0.001), glucose (OR = 1.007, 95% CI: 1.002–1.012, *p* = 0.005), and creatinine (OR = 20.062, 95% CI: 5.564–72.343, *p* < 0.001) were independently associated with revascularization (Table 3 and Table 4, respectively). However, NPS was not associated with revascularization significantly. These findings suggest that a higher NPS is significantly correlated with the presence of HRP in patients with chronic coronary syndrome. Prior to performing multivariable logistic regression, we assessed multicollinearity among the candidate predictors. All variables included in the final models demonstrated acceptable collinearity, with variance inflation factor (VIF) values < 2.0 and no pairwise correlation coefficients exceeding 0.70. These findings confirmed the suitability of the variables for inclusion in multivariable analysis. The AUC for the multivariable model predicting HRP was 0.730 (95% CI: 0.691–0.769), indicating good discriminative ability. Similarly, the AUC for the revascularization model was 0.756 (95% CI: 0.710–0.801).

## 4. Discussion

This study investigated the association between the NPS, a marker of systemic inflammation and nutritional status, and the presence of HRP in patients with CCS undergoing CCTA. Our findings demonstrate that a higher NPS is significantly associated with the presence of HRP, highlighting the potential role of nutritional and inflammatory status in coronary plaque vulnerability. The relationship between systemic inflammation and atherosclerosis progression has been well established [12,13]. Inflammatory markers, such as the NLR and CRP, have been implicated in plaque instability and cardiovascular events [6,14]. In our study, patients with higher NPS exhibited significantly elevated CRP levels and white blood cell counts, supporting the hypothesis that inflammation contributes to HRP formation. Additionally, lower albumin levels were observed in patients with HRP, consistent with prior studies linking hypoalbuminemia to adverse cardiovascular outcomes [15,16]. Nutritional status is an increasingly recognized factor in cardiovascular disease prognosis. The NPS, which integrates albumin levels, total cholesterol, NLR, and LMR, was originally designed to assess cancer-related outcomes but has gained attraction in cardiovascular research [6,7,8]. The selection of the NPS in this study was based on its unique integration of systemic inflammation and nutritional status—two key factors that have been increasingly linked to the development and progression of atherosclerosis. While the NPS was originally validated in oncologic populations, recent cardiovascular studies have demonstrated its prognostic value in various acute and chronic cardiac settings. Unlike traditional cardiovascular risk scores such as GRACE, SYNTAX, or ASCVD, which focus primarily on hemodynamic status, anatomical complexity, or conventional metabolic parameters, the NPS reflects host-related factors that may contribute to plaque vulnerability. Importantly, our intention was not to suggest NPS as a replacement for these established tools, but rather to explore its potential as a complementary biomarker for risk stratification—particularly in the assessment of high-risk coronary plaques that may not be readily predicted by conventional scoring systems. Further prospective studies are warranted to evaluate how NPS may be integrated into existing risk assessment frameworks.

In our study, the presence of HRP was significantly associated with older age, male sex, hypertension, dyslipidemia, and elevated fasting blood glucose and creatinine levels—findings that align with established cardiovascular risk factors [17]. Multivariable analysis revealed that NPS remained an independent predictor of HRP even after adjusting for these confounders, suggesting its potential utility as a novel risk stratification tool. Furthermore, patients with higher NPS had significantly increased CAC scores, indicating a greater atherosclerotic burden, which has been linked to higher cardiovascular event rates [18]. The clinical implications of these findings are significant. Traditional risk scores, such as the Framingham Risk Score and atherosclerotic cardiovascular disease risk calculator, primarily focus on conventional metabolic and hemodynamic parameters [19]. The addition of NPS to risk assessment strategies may provide a more comprehensive evaluation by integrating inflammatory and nutritional factors, thereby improving the prediction of high-risk plaque presence and cardiovascular events. Additionally, our study highlights the importance of addressing malnutrition and systemic inflammation as potential modifiable targets in cardiovascular prevention strategies. Although revascularization was included as an outcome in this study, it represents a surrogate endpoint that is subject to clinical judgment and institutional variation, rather than a direct measure of disease progression or adverse cardiovascular events. In our multivariable analysis, the NPS was no longer an independent predictor of revascularization. This highlights the complexity of interpreting revascularization decisions, which may not fully reflect underlying plaque vulnerability or systemic risk. As such, our primary outcome remains the presence of HRP on CCTA, a validated imaging marker associated with future cardiovascular events. Further prospective studies with longitudinal follow-up and hard clinical endpoints (e.g., myocardial infarction, death, hospitalization) are warranted to more comprehensively evaluate the prognostic role of NPS in chronic coronary syndrome.

Despite these findings, this study has several limitations. First, the retrospective, single-center design inherently introduces the risk of selection bias and limits the generalizability of our findings to broader populations. Additionally, while revascularization decisions were made according to current clinical guidelines, they were ultimately physician-driven and may have been influenced by subjective clinical judgment, availability of additional diagnostic information, or institutional practices. This introduces the potential for treatment selection bias, which could affect the observed association between NPS and revascularization outcomes. Moreover, the lack of standardized criteria for intervention across all cases may limit the reproducibility of our findings in other clinical settings. These factors should be taken into consideration when interpreting the results, and future prospective, multicenter studies are warranted to validate the predictive value of NPS in more diverse populations and under controlled decision-making environments. Second, the role of cardiovascular medications in modulating systemic inflammation and lipid levels is an important consideration in interpreting our results. Statins, in particular, have been shown to reduce LDL-C, improve endothelial function, and exert anti-inflammatory effects, all of which may influence both the components of the NPS and coronary plaque characteristics. For this reason, patients on lipid-lowering therapy at the time of enrollment were excluded to minimize confounding in NPS calculation. However, the potential impact of other medications—including antiplatelet agents, ACE inhibitors/ARBs, and non-steroidal anti-inflammatory drugs (NSAIDs)—on systemic inflammation and plaque vulnerability must also be considered. While these agents were not used as exclusion criteria, they may have influenced levels of inflammatory markers such as NLR or LMR, thereby affecting NPS and possibly plaque morphology. This represents a limitation of our study and underscores the need for future prospective research to account for the influence of pharmacologic therapies on both inflammatory status and coronary plaque features. The exclusion of patients receiving lipid-lowering therapy may have influenced the observed lipid profile distributions. Lastly, the use of CCTA to identify HRP, although highly effective, may not fully capture all aspects of plaque vulnerability that could be assessed by intravascular imaging modalities such as optical coherence tomography or intravascular ultrasound.

## 5. Conclusions

In this study, we demonstrated a significant association between the NPS—a composite index reflecting systemic inflammation and nutritional status—and the presence of HRP identified by coronary computed tomography angiography in patients with chronic coronary syndrome. Our findings suggest that NPS may serve as a valuable complementary tool in cardiovascular risk stratification, providing additional insight into plaque vulnerability beyond traditional metabolic and anatomical risk factors. Although NPS was not an independent predictor of revascularization in the multivariable analysis, its association with HRP supports its potential role in identifying patients with subclinical plaque instability who may be at higher risk of future cardiovascular events. These results highlight the need to consider systemic inflammation and nutritional status as modifiable components of residual cardiovascular risk. Given the limitations of our retrospective, single-center design and the absence of long-term clinical outcome data, prospective multicenter studies with standardized decision-making criteria and follow-up for major adverse cardiovascular events are warranted to validate the prognostic utility of NPS in broader patient populations.

## Figures and Tables

**Table 1:** Baseline characteristics of the patients according to the presence or absence of high-risk coronary plaques.

		High-Risk Plaque Present (*n* = 220)	High-Risk Plaque Absent (*n* = 533)	Total (*n* = 753)	*p* Value
Age		61.7 ± 9.9	54.9 ± 10.3	56.9 ± 10.7	<0.001
Gender (Female), *n* (%)		55 (25)	246 (46.2)	301 (40)	<0.001
Smoking, *n* (%)		66 (30)	173 (32.5)	239 (31.7)	0.510
Diabetes mellitus, *n* (%)		48 (21.8)	95 (17.8)	143 (19)	0.204
Hypertension, *n* (%)		90 (40.9)	154 (28.9)	244 (32.4)	0.001
Dyslipidemia, *n* (%)		32 (14.5)	46 (8.6)	78 (10.4)	0.015
Chronic obstructive pulmonary disease, *n* (%)		38 (17.3)	90 (16.9)	128 (17)	0.898
Left ventricular ejection fraction, %		62 (45–67)	63 (50–69)	62 (45–69)	0.001
Fasting blood glucose, mg/dL		102 (74–372)	98 (67–396)	99 (67–396)	0.002
Creatinine, mg/dL		0.9 (0.5–1.8)	0.8 (0.5–1.3)	0.9 (0.5–1.8)	<0.001
ALT, units/L		18 (8–87)	19 (3–118)	19 (3–118)	0.072
AST, units/L		20 (4–66)	20 (6–131)	20 (4–131)	0.930
Total cholesterol, mg/dL		187.2 ± 46	190 ± 43	189.5 ± 43.8	0.357
LDL-C, mg/dL		117.1 ± 33.5	120 ± 30.7	119.1 ± 31.5	0.252
HDL-C, mg/dL		42 (17–86)	44 (22–95)	44 (17–95)	<0.001
Triglyceride, mg/dL		124.5 (46–1344)	130 (31–625)	130 (31–1344)	0.938
Hemoglobin, g/dL		13.8 ± 1.7	13.8 ± 1.8	13.8 ± 1.6	0.994
White blood cell count, 10^3^/µL		7.6 ± 1.8	7.5 ± 1.8	7.6 ± 1.8	0.813
Platelet count, 10^3^/µL		238 (107–509)	248 (53–632)	246 (53–632)	0.037
CRP, mg/dL		1.9 (0.1–16)	1.8 (0.1–26.5)	1.9 (0.1–26.5)	0.568
Albumin, g/dL		4.1 ± 0.3	4.2 ± 0.2	4.1 ± 0.3	<0.001
ACEI/ARBs, *n* (%)		81 (36.8)	140 (26.3)	221 (29.3)	0.004
Calcium channel blockers, *n* (%)		38 (17.3)	69 (12.9)	107 (14.2)	0.122
Diuretics, *n* (%)		52 (23.6)	92 (17.3)	144 (19.1)	0.043
Beta-blockers, *n* (%)		25 (11.4)	38 (7.1)	63 (8.4)	0.056
Antiplatelet, *n* (%)		52 (23.6)	82 (15.4)	134 (17.8)	0.007
Anticoagulant, *n* (%)		8 (3.6)	14 (2.6)	22 (2.9)	0.454
CACS		99.5 (0–2521)	0 (0–1576)	1 (0–2521)	<0.001
Revascularization, *n* (%)		109 (49.5)	10 (1.9)	119 (12.9)	<0.001
NPS		1.5 ± 0.9	1.2 ± 0.9	1.3 ± 0.9	0.001
NPS group, *n* (%)	1	23 (10.5)	105 (19.7)	128 (17)	
	2	169 (76.8)	381 (71.5)	550 (73)	0.004
	3	28 (12.7)	47 (8.8)	75 (10)	

ALT: alanine transaminase, AST: aspartate transaminase, LDL-C: low-density lipoprotein cholesterol, HDL-C: high-density lipoprotein cholesterol, CRP: C-reactive protein, CACS: coronary artery calcium score, NPS: Naples Prognostic Score.

**Table 2:** Baseline characteristics of the patients according to the Naples Prognostic Score groups.

	NPS Group 1 (*n* = 128)	NPS Group 2 (*n* = 550)	NPS Group 3 (*n* = 75)	Total (*n* = 753)	*p* Value
Age	56.4 ± 8.6	56.3 ± 10.9	61.4 ± 11	56.9 ± 10.7	0.001
Gender (Female), *n* (%)	72 (56.3)	203 (36.9)	26 (34.7)	301 (40)	<0.001
Smoking, *n* (%)	34 (26.6)	187 (34)	18 (24)	239 (31.7)	0.084
Diabetes mellitus, *n* (%)	31 (24.2)	98 (17.8)	14 (18.7)	143 (19)	0.250
Hypertension, *n* (%)	35 (27.3)	174 (31.6)	35 (46.7)	244 (32.4)	0.014
Dyslipidemia, *n* (%)	17 (13.3)	53 (9.6)	8 (10.7)	78 (10.4)	0.474
Chronic obstructive pulmonary disease, *n* (%)	25 (19.5)	88 (16)	15 (20)	128 (17)	0.484
Left ventricular ejection fraction, %	62 (55–69)	63 (45–68)	61 (50–66)	62 (45–69)	0.059
Fasting blood glucose, mg/dL	98.5 (72–396)	99 (67–359)	103 (72–372)	99 (67–396)	0.406
Creatinine, mg/dL	0.8 (0.5–1.3)	0.9 (0.5–1.5)	0.9 (0.5–1.8)	0.9 (0.5–1.8)	0.027
ALT, units/L	21.5 (8–59)	18 (3–118)	17 (7–76)	19 (3–118)	0.046
AST, units/L	21 (10–51)	20 (6–131)	20 (4–53)	20 (4–131)	0.153
Total cholesterol, mg/dL	230.2 ± 37.1	184 ± 40.8	160.5 ± 28.4	189.5 ± 43.8	<0.001
LDL-C, mg/dL	142.4 ± 28.2	116 ± 30.2	99.1 ± 24.8	119.1 ± 31.5	<0.001
HDL-C, mg/dL	45 (25–93)	44 (17–95)	40 (22–75)	44 (17–95)	0.001
Triglyceride, mg/dL	147 (55–1344)	127 (31–625)	112 (38–411)	130 (31–1344)	<0.001
Hemoglobin, g/dL	14 ± 1.3	13.8 ± 1.6	13.2 ± 1.7	13.8 ± 1.6	0.004
White blood cell count, 10^3^/µL	7.2 ± 1.6	7.6 ± 1.9	8 ± 1.6	7.6 ± 1.8	0.021
Platelet count, 10^3^/µL	254 (126–593)	243 (53–632)	241 (72–485)	246 (53–632)	0.042
CRP, mg/dL	1.9 (0.1–8.7)	1.8 (0.1–26.5)	2.1 (0.1–17.5)	1.9 (0.1–26.5)	0.021
Albumin, g/dL	4.2 ± 0.2	4.1 ± 0.3	3.9 ± 0.3	4.1 ± 0.3	<0.001
ACEI/ARBs, *n* (%)	31 (24.2)	159 (28.9)	31 (41.3)	221 (29.3)	0.032
Calcium channel blockers, *n* (%)	13 (10.2)	74 (13.5)	20 (26.7)	107 (14.2)	0.003
Diuretics, *n* (%)	24 (18.8)	98 (17.8)	22 (29.3)	144 (19.1)	0.059
Beta-blockers, *n* (%)	8 (6.3)	47 (8.5)	8 (10.7)	63 (8.4)	0.525
Antiplatelet, *n* (%)	16 (12.5)	99 (18)	19 (25.3)	134 (17.8)	0.068
Anticoagulant, *n* (%)	1 (0.8)	16 (2.9)	5 (6.7)	22 (2.9)	0.056
CACS	0 (0–1576)	1 (0–2521)	21 (0–2093)	1 (0–2521)	<0.001
Revascularization, *n* (%)	16 (12.5)	81 (14.7)	22 (29.3)	119 (15.8)	0.003

NPS: Naples Prognostic Score, ALT: alanine transaminase, AST: aspartate transaminase, LDL-C: low-density lipoprotein cholesterol, HDL-C: high-density lipoprotein cholesterol, CRP: C-reactive protein, CACS: coronary artery calcium score.

**Table 3:** Univariable and multivariable logistic regression analyses investigating the factors related to high-risk coronary plaques.

Univariable	*p* Value	OR	95% CI		Multivariable	*p* Value	OR	95% CI	
			Lower	Upper				Lower	Upper
Age	<0.001	1.067	1.049	1.084	Age	<0.001	1.056	1.038	1.075
Hypertension	0.001	1.704	1.228	2.364	Hypertension	-	-	-	-
Dyslipidemia	0.017	1.802	1.113	2.917	Dyslipidemia	0.032	1.831	1.052	3.185
Diabetes mellitus	0.204	1.287	0.872	1.899	Diabetes mellitus	0.032	1.788	1.051	3.040
Glucose	0.002	1.006	1.002	1.009	Glucose	0.001	1.008	1.003	1.013
Creatinine	<0.001	21.690	7.911	59.467	Creatinine	<0.001	11.005	3.734	32.432
NPS	0.001	1.350	1.133	1.609	NPS	0.036	1.228	1.013	1.489

OR: odds ratio, NPS: Naples Prognostic Score.

**Table 4:** Univariable and multivariable logistic regression analyses investigating the factors related to revascularization.

Univariable	*p* Value	OR	95% CI		Multivariable	*p* Value	OR	95% CI	
			Lower	Upper				Lower	Upper
Age	<0.001	1.067	1.046	1.089	Age	<0.001	1.050	1.026	1.074
Hypertension	0.001	2.023	1.358	3.014	Hypertension	-	-	-	-
Dyslipidemia	<0.001	2.713	1.601	4.598	Dyslipidemia	0.001	2.787	1.505	5.161
Diabetes mellitus	0.018	1.733	1.101	2.729	Diabetes mellitus	-	-	-	-
Glucose	0.001	1.006	1.003	1.010	Glucose	0.005	1.007	1.002	1.012
Creatinine	<0.001	34.788	10.707	113.030	Creatinine	<0.001	20.062	5.564	72.343
NPS	0.004	1.366	1.102	1.694	NPS	-	-	-	-

OR: odds ratio, NPS: Naples Prognostic Score.

## Data Availability

The original contributions presented in this study are included in the article. Further inquiries can be directed to the corresponding author.

## Abbreviations

CAC	Coronary artery calcification
CAD	Coronary artery disease
CCS	Chronic coronary syndrome
CCTA	Coronary CT angiography
HRP	High-risk coronary plaques
HU	Hounsfield units
LMR	Lymphocyte-to-monocyte ratio
NLR	Neutrophil-to-lymphocyte ratio
NPS	Naples Prognostic Score
NRS	Napkin-ring sign
SC	Spotty calcification
